# Cigarette smoking-induced acute eosinophilic pneumonia: A case report

**DOI:** 10.1097/MD.0000000000014704

**Published:** 2019-03-01

**Authors:** Xing Liu, Wangyuan Sun, Wenshu Meng, Yonglong Xiao, Ganzhu Feng, Bin Shi

**Affiliations:** a Department of Respiratory Medicine, The Affiliated Suqian Hospital of Xuzhou Medical University, Suqian; b Department of Respiratory Medicine, The Affiliated Drum Tower Hospital of Nanjing University Medical School, Nanjing; c Department of Respiratory Medicine, The Second Affiliated Hospital of Nanjing Medical University, Nanjing, China.

**Keywords:** acute eosinophilic pneumonia, bronchoalveolar lavage, smoking

## Abstract

**Rationale:**

Acute eosinophilic pneumonia (AEP) is a rare pulmonary disease, which is characterized by diffuse pulmonary eosinophilia. The pathogenesis remains unknown. Here we report a patient with AEP following a recently acquired habit of smoking.

**Patient concerns:**

A 21-year-old female presented with fever, dry cough, and acute hypoxic respiratory distress for 2 days. Chest computed tomography showed bilateral ground glass opacities, patchy nodules, and pleural effusions. Blood tests showed a gradually raised peripheral eosinophils level.

**Diagnoses:**

Bronchoalveolar lavage fluid revealed marked elevation of eosinophils. She was diagnosed with AEP.

**Interventions:**

Systemic methylprednisolone was immediately used for treatment.

**Outcomes:**

Her clinical symptoms and chest radiographs improved promptly after treatment.

**Lessons:**

Cigarette smoking might be an underlying triggering factor of AEP. Diffuse alveolar infiltrates and a gradually increasing peripheral eosinophilia should raise the concern especially in recent smoking patients.

## 1 Introduction

Acute eosinophilic pneumonia (AEP) is characterized by acute fever, hypoxic respiratory failure, diffuse pulmonary eosinophilia, and a rapid therapeutic response to corticosteroid without relapse.^[1,2]^ The etiology remains unknown; however, as reported in previous articles, cigarette smoking may be a potential trigger.^[2–5]^ Diffuse alveolar infiltrates are the most common radiographic findings.^[6]^ Laboratory examinations always show neutrophil predominance and severe hypoxemia; only a few patients have peripheral eosinophilia.^[2]^ As the clinical history is similar to acute interstitial pneumonitis, acute hypersensitivity pneumonitis, and acute respiratory distress syndrome, it is easily misdiagnosed.^[7]^ Corticosteroid remains the mainstay of treatment and no recurrence has been reported.^[1,8,9]^ We present here a case of AEP following a recently acquired habit of smoking.

## 2 Method

We obtained the patient's medical records and reviewed the related literature. The patient has provided informed consent. This case report is not a clinical trial and just incidental interventional process, so ethical approval was not necessary.

## 3 Case report

**Figure 1:**
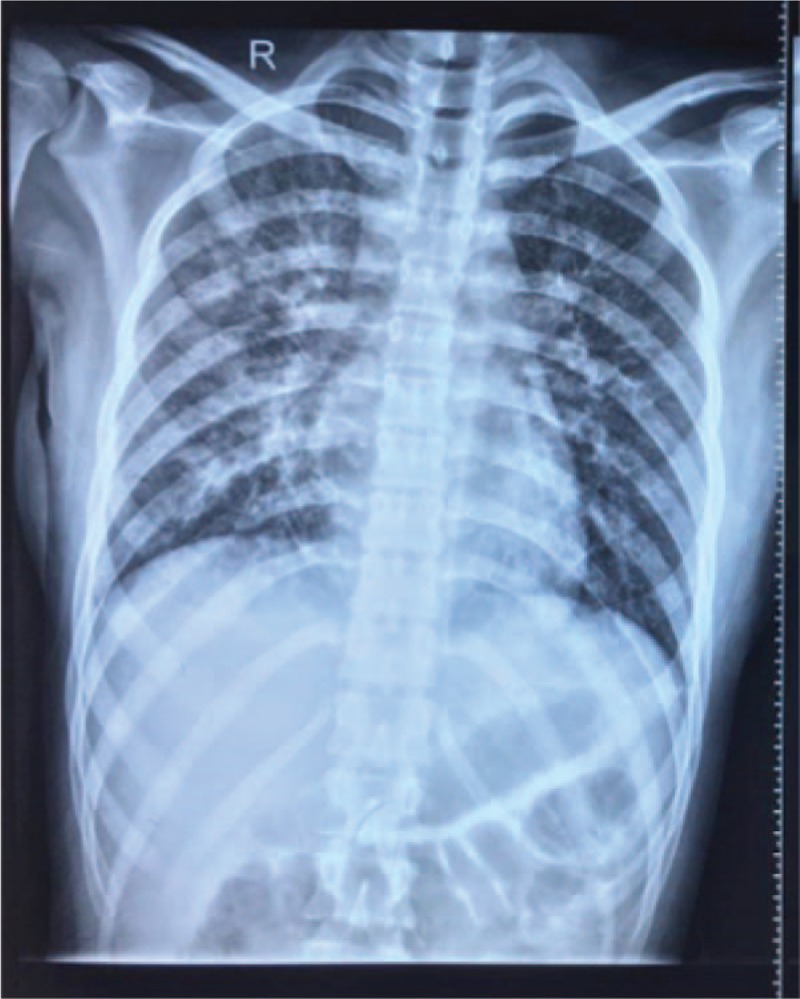
Chest x-ray at local hospital showed bilateral diffuse infiltrations.

A 22-year-old female college student was admitted to the local hospital because of high fever, dry cough, and dyspnea for 2 days. Chest x-ray showed bilateral infiltrates (Fig. 1). Intravenous antibiotics were given immediately for suspected community-acquired pneumonia, but the fever was uncontrolled and the dyspnea rapidly progressed. She was subsequently sent to our department.

**Figure 2:**
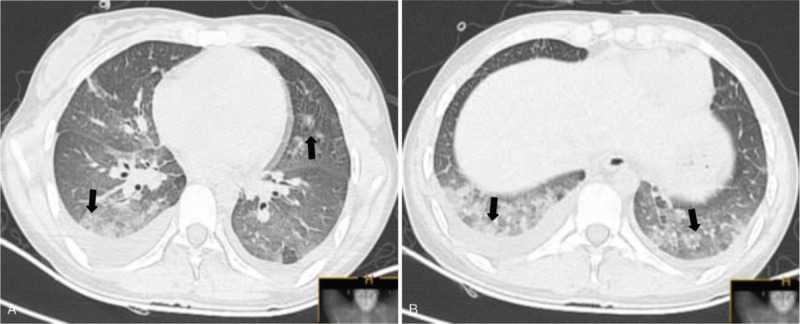
(A, B) Chest computed tomography scan on admission revealed bilateral ground glass opacities, patchy nodular, and pleural effusions.

She denied any history of drug use, insect bite, or travel history recently, but became a passive smoker about 2 months and started smoking 3 to 5 cigarettes per day for 5 days before presentation. On admission, she was febrile, with temperature of 38.5°C. The pulse rate was 105 beat/min, blood pressure was 108/63 mmHg, respiratory rate was 25 breaths/min, and oxygen saturation was 91% while on 2 L oxygen via nasal cannula. The patient had mild respiratory distress. The lips were cyanotic. Chest auscultation showed bilateral crackles. The remainder physical examination was unremarkable. The laboratory investigations showed a white blood cell (WBC) count of 23.92 × 10^9^ cells/L, neutrophils of 22.09 × 10^9^ cells/L, lymphocytes of 0.52 × 10^9^ cells/L, and eosinophils of 0.59 × 10^9^ cells/L. Serum total IgE, C-reactive protein (CRP), and procalcitonin were 192.80 IU/mL (normal, <100IU/mL), 96.95 mg/L and 0.59 ng/mL, respectively. Serum electrolyte, renal and liver functions were normal. Arterial blood gas analysis while breathing ambient air showed a pH of 7.442, PaO_2_ of 49.5 mmHg, PaCO_2_ of 30.4 mmHg. No bacteria was Cultured in the sputum. N-terminal brain-type natriuretic peptide (NT-pro BNP) and left ventricular systolic function on echocardiography were also unremarkable. Chest CT scans revealed bilateral ground glass opacities, patchy nodules, and pleural effusions (Fig. 2A, B).

**Figure 3:**
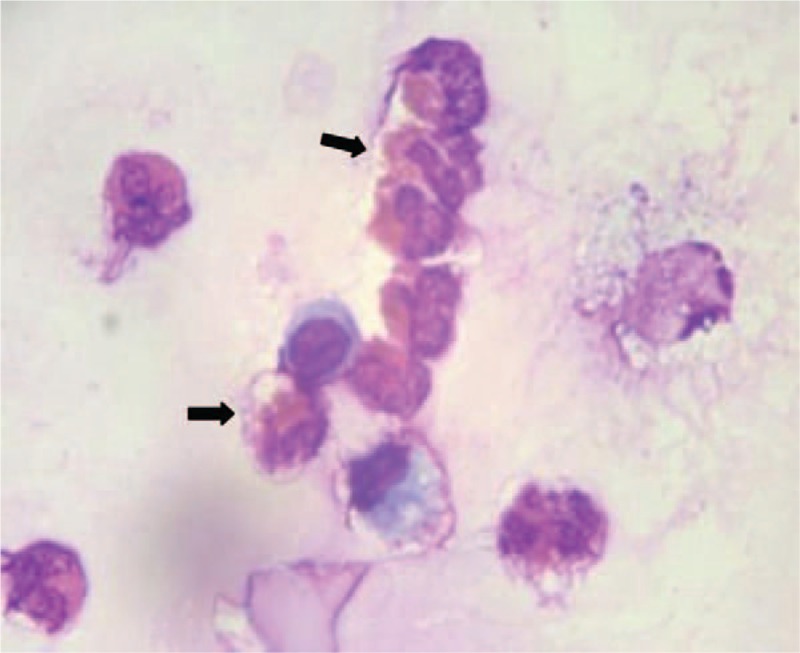
Bronchoalveolar lavage fluid showed a markedly increased percentage of eosinophils.

**Figure 4:**
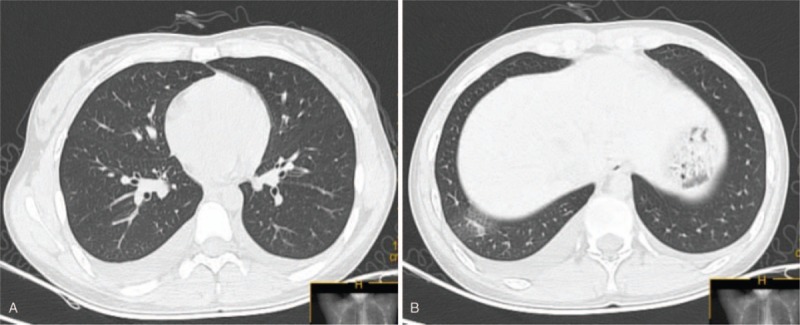
(A, B) Chest computed tomography showed improvement in bilateral infiltrates after 4 days methylprednisolone treatment.

**Figure 5:**
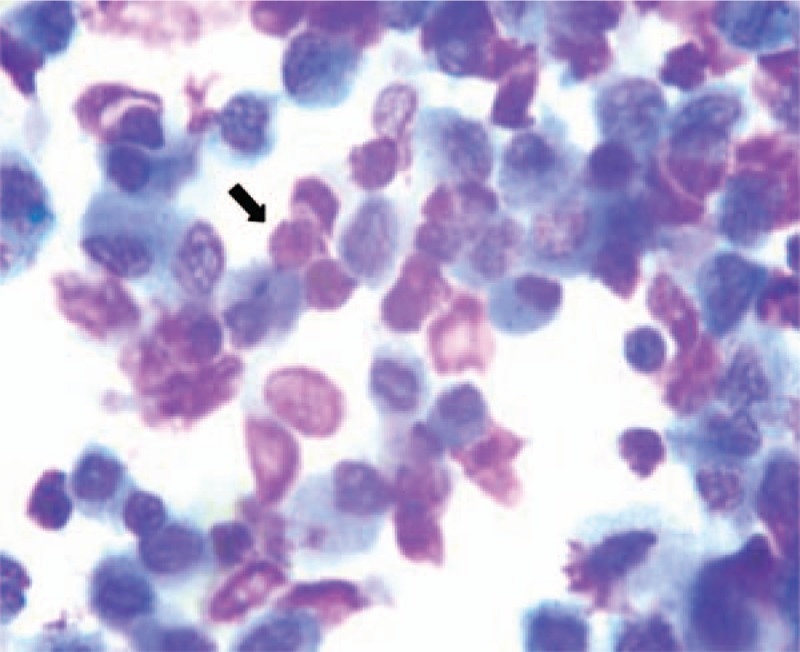
Bronchoalveolar lavage showed a significant decrease of eosinophils after 1-week course of methylprednisolone therapy.

She was started empirically on imipenem and linezolid for presumed severe pneumonia. Further detection revealed normal antineutrophil cytoplasmic antibodies and anti-nuclear antibody. Antigen-specific IgM against Epstein–Barr virus, cytomegalovirus, coxsackie virus were all negative. Pulmonary functional tests suggested decreased diffusion function. Repeat examinations showed CRP, WBC count, and neutrophils were within normal range, but the eosinophil increased to 0.98 × 10^9^ cells/L. Subsequently, she underwent bronchoscopy with bronchoalveolar lavage (BAL). Bronchoscopy showed mucosal inflammation of the trachea, whereas the BAL fluid revealed approximately 50% eosinophils, 3% lymphocytes, 22% macrophages, 2% neutrophils (Fig. 3). We did not find malignant cells and no bacteria, mycobacterium, or fungus were cultured in the BAL fluid. Based on her clinical history, BAL, and typical radiologic findings, she was diagnosed with AEP. Systemic methylprednisolone therapy was immediately used for treatment. Her clinical condition improved dramatically. Repeat arterial blood gas analysis showed pH of 7.411, PaO2 of 108.3 mmHg, and PaCO2 of 36 mmHg. After 4 days, the peripheral eosinophil decreased to 0.14 × 10^9^ cells/L. Chest CT showed rapidly resolution of the infiltration (Fig. 4A, B). Pulmonary functional tests were normal. A second BAL showed a significant decrease of eosinophils (24%) in BAL fluid (Fig. 5). Eventually, she was discharged with oral low dose of steroids. About 1 month later, her peripheral eosinophils level decreased to normal, and chest CT revealed no abnormal findings, with only a slight increase of serum total IgE. At the 6-months’ follow-up, she has quit smoking with no recurrence.

## 4 Discussion

AEP is an uncommon pulmonary disease that can lead to life-threatening respiratory failure. The concept was first proposed by Allen et al in 1989.^[1]^ It is characterized by rapid progression of chest radiographs, diffuse pulmonary eosinophilia, and no recurrence. The average age of the patients is generally <30 years.^[1,2]^ The criteria for diagnosis of AEP are as follows: acute onset of febrile respiratory disease (<1 month); bilateral diffuse infiltrates on chest radiography; hypoxemia; lung eosinophilia (either >25% eosinophils in BAL, or marked eosinophilic pneumonia on lung biopsy); no other causes of eosinophilic pneumonia.^[2,4,10]^ Our patient met this diagnostic criteria sufficiently.

The etiology of AEP remains unclear. Previous studies have reported that inhaling a variety of substances could trigger AEP.^[11–13]^ Substantial numbers of patients may have allergic rhinitis before onset. An increasing number of discoveries suggested that changes in smoking habits are predisposing factors, especially among new smokers.^[4,5]^ Our patient had a history of passive smoking and started smoking for 5 days before onset.

The prominent symptoms of AEP are acute fever, dry cough, dyspnea, and progressive hypoxemic respiratory failure.^[2]^ Chest x-ray examination usually shows bilateral infiltrates. Characteristic thin-section CT scans mainly include bilateral diffuse of ground-glass attenuation, centrilobular nodules, thickening of bronchovascular bundle and interlobular septal, and pleural effusion.^[2,6]^ Moreover, the anatomical distribution and zonal dominance of abnormal CT findings are usually random.^[6]^ AEP is often misdiagnosed as severe pneumonia and acute interstitial pneumonia due to radiologic findings. Acute hypersensitivity pneumonitis and alveolar hemorrhage should also be taken into account as differential diagnoses.^[7]^

Only one-third of AEP patients have peripheral eosinophilia.^[14]^ A gradually increasing peripheral eosinophils should raise the concern especially in recent smoking patients.^[3]^ In our case, the percentage of peripheral eosinophils on admission was 0.59 × 10^9^ cells/L, but increased gradually to 0.98 × 10^9^ cells/L. Elevated serum IgE level was also seen in some cases. Pulmonary parenchymal infiltrate with eosinophils was the major diagnostic hallmark of AEP.^[4,5,15]^ Therefore, transbronchial lung biopsy (TBLB) and BAL should be performed as soon as possible. We found infiltration of eosinophils in BAL fluid, but normalized in bronchial wall specimens from bronchial biopsy. It is also significant to exclude other diseases that can lead to pulmonary eosinophilia, such as allergic bronchopulmonary aspergillosis, vasculitis, and parasitic infection.

Generally, the prognosis of AEP is favorable if treated promptly and appropriately. Corticosteroid currently remains the mainstay of treatment for AEP patients.^[8,9]^ Previous studies have shown that radiographic changes began to improve 4 to 11 days after corticosteroids treatment, and without relapses.^[9,15,16]^ A rapid response to corticosteroids therapy was observed in our case. Eventually, she was discharged with oral low dose of corticosteroids and returned for follow-up; the chest radiograph revealed no abnormal findings.

In conclusion, our report presents a case of AEP produced by cigarette smoking. It is characterized by acute fever, severe hypoxemia, and diffuse pulmonary infiltrates on chest imaging. Owing to similarity in clinical symptoms and radiological findings, it is often misdiagnosed in daily clinical practice. Crucial for the diagnosis are the revelation of pulmonary eosinophilia in the BAL fluid and the elimination of other diseases. Diffuse pulmonary infiltrates and a gradually increasing peripheral eosinophils should raise our concern especially in recent smoking patients.

## Author contributions

**Conceptualization:** Xing Liu, Yonglong Xiao.

**Investigation:** Yonglong Xiao, Bin Shi.

**Methodology:** Wangyuan Sun, Wenshu Meng, Bin Shi.

**Resources:** Xing Liu, Wangyuan Sun.

**Validation:** Bin Shi.

**Writing – original draft:** Xing Liu.

**Writing – review & editing:** Ganzhu Feng.
